# CTX-M-65 Extended-Spectrum β-Lactamase–Producing *Salmonella enterica* Serotype Infantis, United States^1^

**DOI:** 10.3201/eid2412.180500

**Published:** 2018-12

**Authors:** Allison C. Brown, Jessica C. Chen, Louise K. Francois Watkins, Davina Campbell, Jason P. Folster, Heather Tate, Jamie Wasilenko, Christine Van Tubbergen, Cindy R. Friedman

**Affiliations:** Centers for Disease Control and Prevention, Atlanta, Georgia, USA (A.C. Brown, J.C. Chen, L.K. Francois Watkins, D. Campbell, J.P. Folster, C. Van Tubbergen, C.R. Friedman);; Food and Drug Administration, Laurel, Maryland, USA (H. Tate);; US Department of Agriculture, Athens, Georgia, USA (J. Wasilenko)

**Keywords:** antibacterial agents, antimicrobial resistance, beta-lactamases, drug resistance, bacteria, poultry, Salmonella, extended-spectrum β-lactamase–producing, ESBL, CTX-M-65, serotype Infantis, travel, treatment failure, United States, Salmonella enterica

## Abstract

Extended-spectrum β-lactamases (ESBLs) confer resistance to clinically important third-generation cephalosporins, which are often used to treat invasive salmonellosis. In the United States, ESBLs are rarely found in *Salmonella*. However, in 2014, the US Food and Drug Administration found *bla*_CTX-M-65_ ESBL-producing *Salmonella enterica* serotype Infantis in retail chicken meat. The isolate had a rare pulsed-field gel electrophoresis pattern. To clarify the sources and potential effects on human health, we examined isolates with this pattern obtained from human surveillance and associated metadata. Using broth microdilution for antimicrobial susceptibility testing and whole-genome sequencing, we characterized the isolates. Of 34 isolates, 29 carried the *bla*_CTX-M-65_ gene with <9 additional resistance genes on 1 plasmid. Of 19 patients with travel information available, 12 (63%) reported recent travel to South America. Genetically, isolates from travelers, nontravelers, and retail chicken meat were similar. Expanded surveillance is needed to determine domestic sources and potentially prevent spread of this ESBL-containing plasmid.

A leading cause of bacterial foodborne disease in the United States is nontyphoidal *Salmonella* (*1*). *Salmonella enterica* serotype Infantis (hereafter called *Salmonella* Infantis) is one of the most common *Salmonella* serotypes in the United States and 1 of 3 serotypes for which incidence has substantially increased (by 60%) in the past 10 years (*2*). *Salmonella* Infantis has been identified in a variety of foods, animals, and environmental settings.

Salmonellosis usually causes a self-limited gastroenteritis; however, current guidelines recommend that antimicrobial therapy be considered for groups of persons at increased risk for invasive infection. For those patients, treatment with ceftriaxone, ciprofloxacin, trimethoprim/sulfamethoxazole, or amoxicillin is recommended (*3*). Extended-spectrum β-lactamases (ESBLs) confer resistance to most third-generation cephalosporins and penicillins, including ampicillin.

Some *Enterobacteriaceae* produce CTX-M ESBLs, which are encoded by *bla*_CTX-M_ genes that were discovered in 1989 (*4*). Since then, their prevalence has increased dramatically (*5*) and they have been isolated worldwide, primarily from *Escherichia coli* (*6*). Identification of ESBLs, including the CTX-M types, in *Salmonella* in the United States is relatively rare (*7*,*8*).

In the United States, the National Antimicrobial Resistance Monitoring System (NARMS) is a surveillance system that tracks changes in the antimicrobial susceptibility of certain enteric bacteria isolated from ill persons, retail meats, and food animals. In July 2015, the US Food and Drug Administration (FDA) notified the Centers for Disease Control and Prevention (CDC) of a CTX-M-65–producing *Salmonella* Infantis strain isolated from retail chicken meat in December 2014 (*9*). The isolate had a rare pulsed-field gel electrophoresis pattern, JFXX01.0787 (pattern 787). To clarify the sources and potential effects of this strain on human health, we analyzed data from several CDC surveillance systems to describe the prevalence, epidemiology, antimicrobial drug resistance, and molecular phylogenetics of *Salmonella* Infantis pattern 787 isolates from humans.

## Methods

### Background Rates

To determine the expected demographics, rates of hospitalization, and international travel among patients with *Salmonella* Infantis infections compared with patients with infections caused by other common nontyphoidal *Salmonella* serotypes, we analyzed data collected through the Foodborne Disease Active Surveillance Network (FoodNet; https://www.cdc.gov/foodnet/index.html) during 2012–2015. Begun in 1996, FoodNet has conducted active, population-based surveillance for culture-confirmed cases of infection caused by 9 pathogens transmitted commonly through food, including *Salmonella*. FoodNet is a collaboration of CDC, 10 state health departments, the US Department of Agriculture Food Safety and Inspection Service (USDA-FSIS), and the FDA. The FoodNet surveillance area includes 15% of the US population; these data are used to estimate the burden of US foodborne illnesses, hospitalizations, and deaths (*1*). We defined other common nontyphoidal *Salmonella* as the top 20 *S. enterica* serotypes (excluding Infantis) isolated from humans: Typhimurium, Enteritidis, Newport, Heidelberg, Javiana, Saintpaul, Montevideo, Agona, Oranienburg, Muenchen, Thompson, Hadar, Braenderup, Derby, I 4,[5],12:i:-, Paratyphi B var. L(+) tartrate+, Blockley, Anatum, Mississippi, and Panama. These 20 serotypes represented 69% of nontyphoiodal *Salmonella* isolates reported to FoodNet in 2015.

### Case Finding

We looked for pattern 787 isolates reported to the National Molecular Subtyping Network for Foodborne Disease Surveillance (PulseNet; https://www.cdc.gov/pulsenet/index.html) through 2017. The PulseNet database contains pulsed-field gel electrophoresis patterns from state and local public health laboratories and food regulatory agencies. Only the first isolate from each patient was included in case counts.

CDC also requested patient data and clinical isolates from state and local public health departments for any case with pattern 787 reported through October 2015. Patient data included age, sex, date of symptom onset, hospitalization, and recent international travel (defined as travel outside of the United States in the 7 days before symptom onset). Isolate data included specimen collection date(s) and source.

### Isolate Characterization

We used the NARMS standard broth microdilution protocol (Sensititer; Thermo Fisher Scientific, Oakwood Village, OH, USA) to determine the MICs for 14 antimicrobial agents: gentamicin, streptomycin, ampicillin, amoxicillin/clavulanic acid, ceftiofur, ceftriaxone, cefoxitin, azithromycin, sulfasoxazole, trimethoprim/sulfamethoxazole, chloramphenicol, ciprofloxacin, nalidixic acid, and tetracycline (*10*). These agents were categorized into 9 classes defined by the Clinical and Laboratory Standards Institute guidelines (https://clsi.org/); where available, CLSI interpretive criteria were used to define resistance. Transformation studies to confirm plasmid-associated genes were conducted by using plasmid purification and electroporation as previously described (*11*).

Whole-genome sequencing was performed according to the PulseNet protocol for the Illumina MiSeq (Illumina, La Jolla, CA, USA) (*12*). Closed PacBio genomes were generated as part of a previous study and used as references where indicated (*8*). Genome assemblies for short-read data were generated de novo and analyzed for acquired antimicrobial drug–resistance determinants and plasmid replicons by using ResFinder and PlasmidFinder (*13*,*14*). To confirm the absence of certain genes, we performed read mapping in CLC Genomics Workbench version 8.5 (https://www.qiagenbioinformatics.com/products/clc-genomics-workbench/). To identify mutational resistance, we extracted the *gyrA* and *parC* genes from genome assemblies by using a perl script (https://github.com/lskatz/lskScripts/blob/master/blastAndExtract.pl). To identify mutations in the quinolone resistance–determining regions of these genes, we aligned gene sequences in CLC Workbench. To assess isolate relatedness, we generated high-quality single-nucleotide polymorphism (hqSNP) phylogenies. In brief, isolates were aligned to the closed chromosomal sequence of 2014AM-3028 (omitting the plasmid contig) by using Lyve-SET-v1.1.4f (https://github.com/lskatz/lyve-SET/). Genome alignments were processed by using Gubbins (https://sanger-pathogens.github.io/gubbins/) to omit areas of recombination, uninformative sites were removed, and the resulting SNP alignment was used to calculate pairwise differences and generate hqSNP trees by using scripts bundled with Lyve-SET (*15*,*16*). We performed a phylogeographic analysis (a type of molecular clock analysis) by using Bayesian Evolutionary Analysis Sampling Trees (BEAST; https://journals.plos.org/ploscompbiol/article?id=10.1371/journal.pcbi.1003537). 

To sample more diverse *Salmonella* Infantis isolates, we obtained sequence data on isolates from sources other than CDC NARMS assigned to the same National Center for Biotechnology Information (NCBI) SNP cluster PDS000003955.192 as our study isolates on the NCBI Pathogen Detection page (*17*). These additional genomes were from isolates recovered from hospitalized patients, meat, and environmental samples from Peru during 2010–2014 and collected by the Center for Food Safety and Applied Nutrition at FDA and the US Naval Medical Research Unit (*18*), and isolates from chicken samples obtained at slaughter by USDA-FSIS. These isolates were used to produce a time-measured maximum clade credibility tree to estimate the dates that study isolates in addition to all isolates (study isolates and isolates from Peru and cecal isolates from USDA-FSIS) shared most recent common ancestors (MRCAs), and the geographic location of these MRCAs (*18*). The date of the MRCA was estimated by reporting the height of the most ancestral node of the maximum clade credibility tree and the 95% highest posterior density (HPD) intervals for these estimates. More details on genetic analyses are available in the Technical Appendix.

### Statistical Analyses

We used the χ^2^ test (or Fisher exact test when cell counts were ≤5) for statistical comparisons. All denominators exclude persons for whom data were missing. We considered p<0.05 to be significant. All p values were 2-tailed. We used SAS 9.3 or 9.4 (SAS Institute, Cary, NC, USA) to conduct our analyses.

## Results

**Figure 1:**
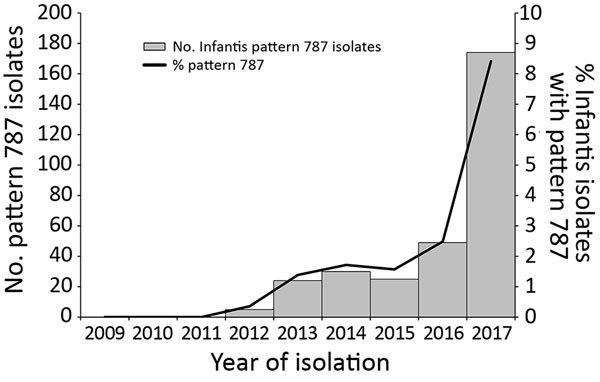
Total of 312 *Salmonella enterica* serotype Infantis isolates from humans with pattern JFXX01.0787 as a percentage of all *Salmonella* Infantis isolates by year, United States, 2012–2017. Source: PulseNet (https://www.cdc.gov/pulsenet/index.html).

The first pattern 787 *Salmonella* Infantis isolate from a human in the United States was collected from a patient in June 2012. By the end of 2017, PulseNet contained 312 *Salmonella* Infantis pattern 787 isolates from persons living in 43 states; Washington, DC; and Puerto Rico. The number of cases detected each year increased from 5 in 2012 to 174 in 2017 and represented 8.4% of all *Salmonella* Infantis isolated that year (Figure 1).

State health departments submitted 34 pattern 787 isolates from humans to CDC; 29 (85%) had resistance phenotypes consistent with ESBL-conferred resistance to ceftriaxone, ceftiofur, and ampicillin (Tables 1, 2). All 29 isolates with ESBL-resistant phenotypes had the *bla*_CTX-M-65_ gene. In addition to ESBL-conferred resistance, these isolates were also resistant to chloramphenicol, sulfisoxazole, tetracycline, nalidixic acid, and trimethoprim/sulfamethoxazole and intermediately susceptible to ciprofloxacin and gentamicin. Resistance to these drugs was plasmid mediated and transferable to *E. coli,* except for nalidixic acid and ciprofloxacin, for which resistance was caused by a chromosomal mutation in *gyrA* (D87Y). Transferable resistance resulted from a large IncFIB-like plasmid containing the resistance genes *aph(3′)-Ic*, *aph (**4**)-Ia*, *aadA1*, *aac (**3**)-IVa*, *bla*_CTX-M-65_, *fosA*, *floR*, *sul1*, *tetA*, and *dfrA14*. These observations are consistent with the organization of these genes on a single IncF1B-like plasmid closed by using long read sequencing, which was completed as part of a previous study by Tate et al., who recently published a further detailed description of this plasmid and a comparative genomics analysis of *bla*_CTX-M-65_–positive IncFIB-like plasmids from *Salmonella* Infantis in the United States (*9*).

**Table 1:** Characteristics of patients and 30 *Salmonella enterica* serotype Infantis pattern JFXX01.0787 isolates associated with CTX-M-65, United States*

Accession no.†	**Year**	**Specimen source**	**Resistance profile**	**Plasmids**	***gyrA***	***parC***	**Hospitalized**	**Recent travel**	**Prolonged infection‡**
SRR2485281	2012	Urine	ACSuTCxTioCip(I)NalCot Gen(I)	IncFIB-like	D87Y	Wt	Yes	Peru	
SRR2485278	2012	Blood	ACSuTCxTioFoxCip(I)NalCotGen(I)	IncFIB-like	D87Y	Wt	No data	Peru	Yes
SRR4019593	2013	Feces	ASuTCxTioCip(I)NalCotGen	IncFIB-like	D87Y	Wt	No data	No data	
SRR4019592	2013	Feces	ACSuTCxTioCip(I)NalCotGen	IncFIB-like	D87Y	Wt	No data	No data	
SRR2485284	2013	Feces	ACSuTCxTioCip(I)NalCot Gen(I)	IncFIB-like	D87Y	Wt	No data	No data	Yes
SRR4025935	2013	Feces	ACSuTCxTioCip(I)Nal	IncFIB-like	D87Y	Wt	No	Peru	
SRR4025936	2013	Feces	ACSuTCxTioCip(I)NalCot	IncFIB-like	D87Y	Wt	No data	Peru	Yes
SRR3178069	2013	Feces	ACSuTCxTioCip(I)NalCot	IncFIB-like	D87Y	Wt	No	Peru	Yes
SRR4019589	2014	Feces	ACSSuTCxTioCip(I)NalCot Gen	IncFIB-like	D87Y	Wt	No	Ecuador	
SRR2407791	2014	Retail chicken meat	ACTCxTioCip(I)NalGen(I)	IncFIB-like	D87Y	Wt	NA	NA	NA
SRR2353201	2014	Feces	ACSSuTCxTioCip(I)NalCot Gen(I)	IncFIB-like	D87Y	Wt	No data	No data	
SRR4025938	2014	Feces	ACSuTCxTioCip(I)NalCot Gen(I)	IncFIB-like	D87Y	Wt	No data	No data	
SRR2485287	2014	Feces	ACSSuTAug(I)CxTioFox Cip(I)NalCotGen(I)	IncFIB-like	D87Y	Wt	No data	No data	Yes
SRR4019601	2014	Feces	ASSuTCxTioCip(I)NalCotGen	IncFIB-like	D87Y	Wt	No data	No data	Yes
SRR4019595	2014	Feces	ACSSuTCxTioCip(I)NalCot	IncFIB-like	D87Y	Wt	No	No travel	
SRR2485288	2014	Feces	ACSSuTCxTioCip(I)NalCot Gen(I)	IncFIB-like	D87Y	Wt	No data	No travel	
SRR2485282	2014	Feces	ACSSuTCxTioCip(I)NalCot Gen(I)	IncFIB-like	D87Y	Wt	No	Peru	Yes
SRR2485286	2014	Feces	ASSuTCxTioCip(I)NalCotGen	IncFIB-like	D87Y	Wt	Yes	Peru	
SRR3178071	2014	Feces	ACSSuTCxTioCip(I)NalCot	IncFIB-like	D87Y	Wt	Yes	Peru	
SRR4019602	2015	Feces	ACSSuTCxTioFox(I)Cip(I)NalCot	IncFIB-like	D87Y	Wt	No	Ecuador	
SRR4019594	2015	Feces	ACSSuTCxTioCip(I)NalCot	IncFIB-like	D87Y	Wt	No data	No data	
SRR3185043	2015	Urine	ACSSuTCxTioCip(I)NalCot	IncFIB-like	D87Y	Wt	No	No data	Yes
SRR4019591	2015	Feces; urine	ACSSuTCxTioCip(I)NalCot	IncFIB-like	D87Y	Wt	Yes	No data	Yes
SRR4019588	2015	Feces	ACSuTCxTioCip(I)NalCot Gen(I)	IncFIB-like, colE	D87Y	Wt	Yes	No travel	Yes
SRR4019587	2015	Feces	ACSSuTCxTioCip(I)Nal Gen(I)	IncFIB-like	D87Y	Wt	No	No travel	
SRR3184311	2015	Feces	ACSSuTCxTioCip(I)NalCot Gen(I)	IncFIB-like	D87Y	Wt	Yes	No travel	Yes
SRR4019590	2015	Feces	ACSuTCxTioCip(I)NalCot	pXuzhou21, IncFIB-like	D87Y	Wt	Yes	No travel	
SRR4019603	2015	Urine	ACSSuTCxTioCip(I)NalCot	IncFIB-like	D87Y	Wt	No	No travel	
SRR3178070	2015	Urine	ACSSuTCxTioCip(I)NalCot	IncFIB-like	D87Y	Wt	Yes	Peru	
SRR4019600	2015	Feces	ACSuTCxTioCip(I)NalCot	IncFIB-like	D87Y	Wt	No	Peru	Yes

*A, ampicillin; Aug, amoxicillin-clavulanic acid; C, chloramphenicol; Cip, ciprofloxacin; Cot, trimethoprim/sulfamethoxazole; Cx, ceftriaxone; Cx, ceftriaxone; Fox, cefoxitin; Gen, gentamicin; (I), intermediate susceptibility; NA, not applicable; Nal, nalidixic acid; S, streptomycin; Su, sulfisoxazole; T, tetracycline; Tio, ceftiofur. †Accession numbers are the sequence read archive run identification numbers from the National Center for Biotechnology Information. ‡Documented illness >2 wk after symptom onset. Blank cells indicate no evidence of prolonged infection (either no additional data were available or additional data indicated no clear evidence of prolonged infection).

**Table 2:** Characteristics of patients and 5 *Salmonella enterica* serotype Infantis pattern JFXX01.0787 isolates without CTX-M-65, United States*

Accession no.†	Year	Specimen source	Resistance profile	Plasmids	*gyrA*	*parC*	Hospitalized	Recent travel
SRR2485280	2012	Feces	No resistance detected	No replicons detected	None	Wt	No	No travel
SRR4025941	2012	Feces	CSuTCip(I)NalCotGen(I)	IncFIB-like	D87Y	Wt	No	Peru, Bolivia, Ecuador, Chile
SRR4019596	2014	Urine	CSSuTFox(I)Cip(I)NalCot	IncFIB-like	D87Y	Wt	No	No data
SRR3185042	2014	Feces	No resistance detected	No replicons detected	None	Wt	Yes	No travel
SRR2485289	2015	Feces	No resistance detected	No replicons detected	None	Wt	No	No data

*A, ampicillin; Aug, amoxicillin-clavulanic acid; C, chloramphenicol; Cip, ciprofloxacin; Cot, trimethoprim/sulfamethoxazole; Cx, ceftriaxone; Fox, cefoxitin; Gen, gentamicin; (I), intermediate susceptibility; Nal, nalidixic acid; S, streptomycin; Su, sulfisoxazole; T, tetracycline; Tio, ceftiofur.  †Accession numbers are the sequence read archive run identification numbers from the National Center for Biotechnology Information.

We focused our analysis on the 29 patients with *Salmonella* Infantis isolates containing the *bla*_CTX-M-65_ gene (hereafter called CTX-M-65 Infantis). We compared patients infected with CTX-M-65 Infantis with patients infected with all strains of *Salmonella* Infantis and with patients infected with common *Salmonella* serotypes other than Infantis. The median age of patients with CTX-M-65 Infantis was 25 years (interquartile range [IQR] 15–50 years), and 69% were female (Table 3). The median age of patients with any strain of *Salmonella* Infantis was 37 years (IQR 12–58 years), and 56% were female. The median age of patients infected with other common *Salmonella* serotypes was 29 years (IQR 6–54 years), and 52% were female.

**Table 3:** Comparison of demographic and clinical characteristics of patients with CTX-M-65–positive isolates with patients infected with all *Salmonella* *enterica* serotype Infantis strains or other common *Salmonella* serotypes, FoodNet, United States, 2012–2015*

Characteristic	CTX-M-65-positive *Salmonella* Infantis, pattern JFXX01.0787, n = 29*				All *Salmonella* Infantis, n = 723†				Other *Salmonella*, n = 21,285‡		
Proportion§	%	p value	Proportion§	%	p value	Proportion§	%	p value
Female sex	20/29	69	Referent		403/722	56	0.16		11,067/21,258	52	0.07
Urine isolation	5/29	17	Referent		66/723	9	0.14		1,076/21,214	5	<0.01
Hospitalization	8/18	44	Referent		199/695	29	0.14		5,951/20,619	29	0.14
International travel	12/19	63	Referent		51/611	8	<0.01		1,431/16,496	9	<0.01

*Patient age range 0–82 y; median age (interquartile range) 25 (15–50) y.  †Patient age range 0–95 y; median age (interquartile range) 37 (12–58) y. ‡Patient age range 0–101 y; median age (interquartile range) 29 (6–54) y. Other *Salmonella* defined as the top 20 *Salmonella enterica* serotypes (excluding Infantis): Typhimurium, Enteritidis, Newport, Heidelberg, Javiana, Saintpaul, Montevideo, Agona, Oranienburg, Muenchen, Thompson, Hadar, Braenderup, Derby, I 4,[5],12:i:-, Paratyphi B var. L(+) tartrate+, Blockley, Anatum, Mississippi, Panama. §Unknowns were excluded from the denominator.

Of 18 patients infected with CTX-M-65 Infantis for whom outcome data were available, 8 (44%) were hospitalized, compared with a hospitalization rate of 29% among patients in both comparison groups (those with Infantis and those with other *Salmonella* serotypes; Table 3). Patients with CTX-M-65 Infantis infection were more likely (17%) to have the organism isolated from urine compared with all patients with *Salmonella* Infantis (9%, p = 0.14) and patients with other common serotypes (5%, p<0.01).

For 20 CTX-M-65 Infantis–infected patients with information about symptom onset date and isolation date(s), *Salmonella* Infantis was recovered from 12 who provided fecal samples >2 weeks after their reported date of symptom onset. A total of 8 patients were still reporting symptoms and had positive cultures >30 days after symptom onset, including 3 patients who sought care and had multiple isolates recovered from 50 days to 8 months after symptom onset.

Twelve (63%) patients with CTX-M-65 Infantis infections reported international travel in the 7 days before symptom onset. All reported travel to South America, 10 to Peru, and 2 to Ecuador (Table 3). Travel was less common among all *Salmonella* Infantis–­­infected patients (7%, p<0.01) and those infected with other common serotypes (8%, p<0.01; Table 3).

**Figure 2:**
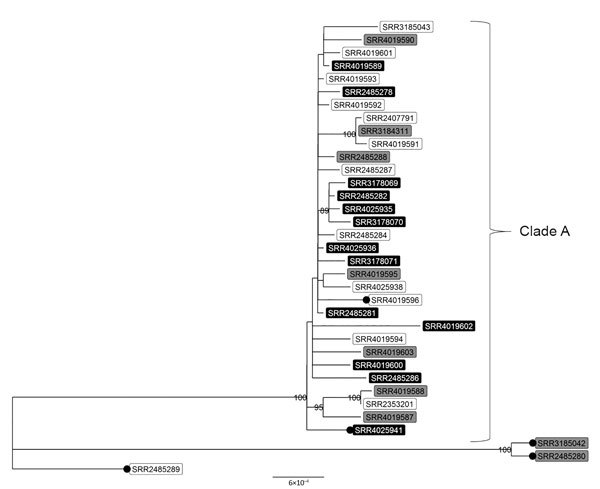
High-quality single-nucleotide polymorphism–based phylogenetic tree of clinical and retail meat isolates of *Salmonella enterica* serotype Infantis with pulsed-field gel electrophoresis pattern JFXX01.0787 collected in the United States and submitted to the National Antimicrobial Resistance Monitoring System for whole-genome sequencing. Tree tips are labeled with National Center for Biotechnology Information accession numbers (sequence read archive run identification numbers); shading indicates patients’ international travel history for clinical isolates (black, recent international travel; gray, no recent international travel; white, travel data missing). Black circles indicate isolates that are missing the *bla*_CTX-M-65_ gene. Isolates in top clade differed by 2–47 high-quality single-nucleotide polymorphisms. Numbers displayed on nodes are bootstrap support values, an indication of the reliability of the tree. Only bootstrap values >80 are displayed. More information on patient and isolate characteristics are provided in Tables 1 and 2. Scale bar indicates nucleotide substitutions per site.

A maximum-likelihood hqSNP phylogeny of the 34 isolates from humans and that from retail chicken meat revealed that isolates from patients with travel-associated infections formed a well-supported clade (clade A) with isolates from patients with infections not associated with travel (Figure 2). Isolates in clade A differed by only 2–47 pairwise hqSNPs, suggesting that these isolates recently evolved from a common ancestor. Included in clade A was the isolate from retail chicken meat collected in 2014.

Of the 5 CTX-M-65-negative isolates, 2 grouped within clade A and contained a multidrug-resistance plasmid that was lacking the *bla*_CTX-M-65_ gene. An additional 3 CTX-M-65–negative isolates lacked the IncFIB-like plasmid replicon, lacked resistance determinants, and differed by 96–273 pairwise hqSNPs from isolates in clade A. Absence of the *bla*_CTX-M-65_ gene in these 5 isolates was confirmed by read-mapping procedures and supported by the observed phenotypic susceptibility to β-lactam antimicrobial drugs.

A phylogeographic analysis of the 32 isolates in the main clade and additional genomes from NCBI generated a time-measured phylogenetic tree of isolates from the United States and Peru (Technical Appendix Figure 1). These isolates last shared a common ancestor around 2006 (95% HPD interval 2003–2008). This analysis also suggests that the MRCA of these isolates existed in Peru with a probability of 98.7%. The probability of the tree being rooted in the United States was ≈1.3%. Clinical isolates from humans in the United States sequenced as part of our study last shared a common ancestor sometime around 2009 (95% HPD interval 2008–2009), before the first isolation of pattern 787 in the United States in 2012.

## Discussion

A new strain of *Salmonella* Infantis, which has pattern 787 and frequently carries a multidrug-resistant plasmid with a CTX-M-65 ESBL, has emerged in the United States. This strain possesses clinically important resistance associated with higher hospitalization rates. Using epidemiologic and phylogenetic methods, we demonstrated that the earliest cases of CTX-M-65 Infantis infection were among travelers returned from South America, whereas subsequent infections were acquired domestically.

The demographic and clinical characteristics among patients with CTX-M-65 Infantis infections differed from those of patients infected with all strains of *Salmonella* Infantis or other common *Salmonella* serotypes. CTX-M-65 Infantis–infected patients were younger and more frequently female, and rates of hospitalization were 50% higher for these patients than for those in the other 2 groups. Among patients with CTX-M-65 Infantis infections, 63% reported recent travel to South America, predominantly to Peru, compared with <10% in the comparison groups reporting travel. This finding is consistent with those of other studies that found foreign travel to be a risk factor for CTX-M–type ESBLs (*19*–*21*). Studies have shown that CTX-M-65 has recently emerged in commensal *E. coli* in Bolivia (*22*) and in *Salmonella* Infantis in Ecuador (*23*), 2 countries to which patients in our study also traveled.

Our phylogeographic analysis, which included many isolates from Peru, suggested that isolates from patients in the United States last shared a common ancestor that existed in Peru in 2009. This finding makes sense, given the high proportion of patients in our study who reported travel to Peru and the known circulation of CTX-M-65–positive *Enterobacteriaceae* in South America. In our study, patients with CTX-M-65 Infantis infection and a history of travel were reported every year from 2012 through 2015; patients with no history of travel were first reported in 2014.

One caveat is that genetic information for CTX-M Infantis from countries in South America other than Peru was not available; therefore, we could not distinguish the role that other countries in South America may have played in the spread of this *Salmonella* Infantis strain to the United States. We cannot definitively determine how patients with this strain of *Salmonella* Infantis who did not report travel to South America became infected; however, our analysis does show a close genetic relationship between clinical isolates from these patients and isolates collected by USDA-FSIS from chickens in the United States.

Poultry consumption may be a source of CTX-M-65 Infantis infection in the United States and abroad. In our study, more than one third of patients with CTX-M-65 Infantis infections did not report recent international travel and thus were exposed via a domestic source. CTX-M genes have been linked to poultry, particularly broiler chickens (*24*–*26*). A study of 14 chicken farms in Henan Province, China, conducted during 2007–2008, was the first to describe detection of CTX-M-65–producing *E. coli* in chickens (*27*). Recently, CTX-M-65 Infantis was found to be transmitted from broiler chickens and chicken meat to humans in Italy (*28*).

The results of our initial hqSNP analysis demonstrated that the original isolate collected from retail chicken in 2014 was genetically related to isolates from humans. In addition, our data show a clear partition between the dates of specimen collection from the first travel-associated infections detected in the United States (2012) and the dates of the first domestically acquired infections detected (2014) (Technical Appendix Figure 2). Sampling in poultry production plants and sequence data from USDA-FSIS demonstrate that this strain was present in domestic food processing plants during the latter part of the study period (2014–2015) (*29*). We cannot, however, determine precisely when or how this strain was introduced into poultry stock in the United States because enhanced poultry plant sampling was not conducted before 2014. International distribution of infected breeder stocks, chicken feed, or feed additives contaminated with CTX-M-65 Infantis could help explain how these broiler-associated infections have spread globally during the same period.

CTX-M-65, in comparison with more well-characterized CTX-M enzymes, differs by only 2 substitutions (A77V, S272R) from CTX-M-14, one of the more commonly detected CTX-M variants worldwide (*30*,*31*). The *bla*_CTX-M-65_ gene was transmitted on a large IncFIB-like plasmid containing multiple resistance genes. The presence of multidrug resistance in CTX-M-65 Infantis isolates in our study suggests that a variety of antimicrobial drugs could provide positive selection pressure and thus promote persistence of this strain. These characteristics suggest that the potential for spread of the gene is high. Research conducted in Bolivia has shown that even in the absence of selective pressure from antimicrobial drug use, plasmid transfer of CTX-M-65 from *E. coli* to other pathogens was achieved at high frequency and shown to be stable (*22*).

Additional studies on antimicrobial drug use and management practices in food animals may help us understand which factors contribute most to the emergence, persistence, and spread of resistance genes such as *bla*_CTX-M-65_. New efforts to perform whole-genome sequencing on all *Salmonella* isolates at public health laboratories nationwide will help determine whether plasmid-mediated *bla*_CTX-M-65_ has spread to other *Salmonella* serotypes. One limitation of our study was that we were unable to obtain epidemiologic data for all 312 cases. The data available from FoodNet enabled us to compare variables such as patient demographics, travel, and hospitalizations; however, we did not have a control population for evaluating other variables of interest to determine potential domestic sources of transmission.

The spread of CTX-M-65 is concerning because the presence of ESBLs eliminates 2 recommended treatment options, ceftriaxone and ampicillin, for the management of salmonellosis. Given the multidrug-resistant profile of CTX-M-65 Infantis, potential for plasmid-mediated transmission, increased hospitalization rate, and evidence of this strain in domestic poultry, action is needed to prevent widespread dissemination in the United States. Enhanced surveillance and additional studies in humans and food animals may help pinpoint the sources of infection for implementation of prevention and control measures. Meanwhile, travelers and healthcare providers should be aware of the risks and implications of infection with this strain, including the potential for antimicrobial treatment failure.

Technical Appendix Supplementary methods, tip-dated maximum clade credibility tree of 160 closely related *Salmonella* serotype Infantis isolates, and dates of specimen collection for isolates in clade A.
