# Supplementary material for: CTX-M-65 Extended-Spectrum β-Lactamase–Producing Salmonella enterica Serotype Infantis, United States
Source: Emerg Infect Dis. 2018 Dec;24(12):2284–91. doi: 10.3201/eid2412.180500 (PMC6256390; doi:10.3201/eid2412.180500)
Supplement: Technical Appendix — Supplementary methods, tip-dated maximum clade credibility tree of 160 closely related Salmonella serotype Infantis isolates, and dates of specimen collection for isolates in clade A. [file 18-0500-Techapp-s1.pdf]

Article DOI: https://doi.org/10.3201/eid2412.180500

CTX-M-65 Extended-Spectrum β-Lactamase–Producing *Salmonella enterica* Serotype Infantis, United States

Technical Appendix

Supplementary Methods

Genomic DNA was extracted using the Qiagen DNeasy Blood and Tissue Kit (Qiagen, Valencia, CA). Paired end, 250 bp whole genome sequencing libraries were generated using the Nextera XT kit and sequenced on the Illumina MiSeq (Illumina, LaJolla CA) according to the PulseNet protocol for Illumina MiSeq (*1*). Genome assemblies were generated de novo using CLC Genomics Workbench v8.5 (Qiagen, Valencia, CA). We used ResFinder v.2.1 and PlasmidFinder v.1.3 with 90 and 50% identity cutoffs, respectively, to identify acquired antimicrobial resistance determinants and plasmid replicons in assembled genomes (*2*,*3*). SNP phylogenies in this study were generated by aligning reads to the closed chromosomal sequence of 2014AM-3028 (NCBI Accession SAMN05255999 [SRR3710049-SRR3710052]) using lyve-SET-v1.1.4f (https://github.com/lskatz/lyve-SET/) and the presets for *Salmonella enterica*. The resulting genome alignment was examined using Gubbins (version released 08–2015) to omit areas of recombination (*4*). The removeUninformativeSites.pl script from Lyve-SET was used to filter out uninformative sites while allowing ambiguities, and the filtered multi-sequence alignment was used to calculate pairwise differences and build high quality single nucleotide polymorphism (hqSNP) trees using the pairwiseDistances.pl and launch_raxml.sh scripts included in Lyve-SET (*5*). The resulting phylogenetic trees were annotated in R v. 3.2.4 with the package "ggtree" (*6*). A discrete Bayesian phylogeography analysis was conducted in in BEAST v. 2.4.7 https://journals.plos.org/ploscompbiol/article?id=10.1371/journal.pcbi.1003537). To sample more diverse Infantis isolates, we obtained sequence data for isolates assigned to the same NCBI SNP cluster (PDS000003955.192) as our study isolates on the NCBI Pathogen Detection page (https://www.ncbi.nlm.nih.gov/pathogens/). Path-O-Gen v. 1.4 was first used to

assess the temporal signal in isolates and thus their suitability for further molecular clock analysis (*7*). The appropriate site model (HKY + Γ + I) was selected using bModelTest (v. 1.0.4) (*8*). The BEAST package MASCOT (v. 0.0.4) was used to conduct the phylogeographic analysis (*9*). Analysis was performed on a filtered SNP matrix detailed above and the output xml file was modified to account for constant sites in the genome (constantSiteWeights = ”1104982 1209720 1202237 1099939”). A strict molecular clock was selected and run for 10^8 iterations, with sampling every 10,000 iterations. Output was evaluated using Tracer v. 1.6. Three independent chains were evaluated and a representative BEAST tree file was used to produce a maximum clade credibility tree using Tree Annotator with default parameters and the “keep” option for heights.

References

1. Centers for Disease Control and Prevention. Laboratory standard operating procedure for PulseNet Nextera XT Library Prep and Run Setup for the Illlumina MiSeq. Code PNL32. 2015 [cited 2016 Feb 1]. https://www.cdc.gov/pulsenet/pdf/pnl32-miseq-nextera-xt.pdf.

2. Zankari E, Hasman H, Cosentino S, Vestergaard M, Rasmussen S, Lund O, et al. Identification of acquired antimicrobial resistance genes. J Antimicrob Chemother. 2012;67:2640–4. PubMed http://dx.doi.org/10.1093/jac/dks261

3. Carattoli A, Zankari E, García-Fernández A, Voldby Larsen M, Lund O, Villa L, et al. In silico detection and typing of plasmids using PlasmidFinder and plasmid multilocus sequence typing. Antimicrob Agents Chemother. 2014;58:3895–903. PubMed http://dx.doi.org/10.1128/AAC.02412-14

4. Croucher NJ, Page AJ, Connor TR, Delaney AJ, Keane JA, Bentley SD, et al. Rapid phylogenetic analysis of large samples of recombinant bacterial whole genome sequences using Gubbins. Nucleic Acids Res. 2015;43:e15. PubMed http://dx.doi.org/10.1093/nar/gku1196

5. Katz LS, Petkau A, Beaulaurier J, Tyler S, Antonova ES, Turnsek MA, et al. Evolutionary dynamics of *Vibrio cholerae* O1 following a single-source introduction to Haiti. MBio. 2013;4:e00398-13. PubMed http://dx.doi.org/10.1128/mBio.00398-13

6. Yu G, Smith D, Zhu H, Guan Y, Lam T. ggtree: an R package for visualization and annotation of phylogenetic tree with their covariates and other associated data. Methods in Ecology and Evolution; 2017;8:28-36.

7. Rambaut AL, Lam TT; Carvalho, LM; Pybus, OG. Exploring the temporal structure of heterochronous sequences using TempEst (formerly Path-O-Gen). Virus Evolution. 2016;2:vew007.

8. Bouckaert RR, Drummond AJ. bModelTest: Bayesian phylogenetic site model averaging and model comparison. BMC Evol Biol. 2017;17:42. PubMed http://dx.doi.org/10.1186/s12862-017-0890-6

9. Mueller NF, Rasmussen DA, Stadler T. MASCOT: Parameter and state inference under the marginal structured coalescent approximation [cited 2018 May]. Bioinformatics. 2018 May 22 [Epub ahead of print].

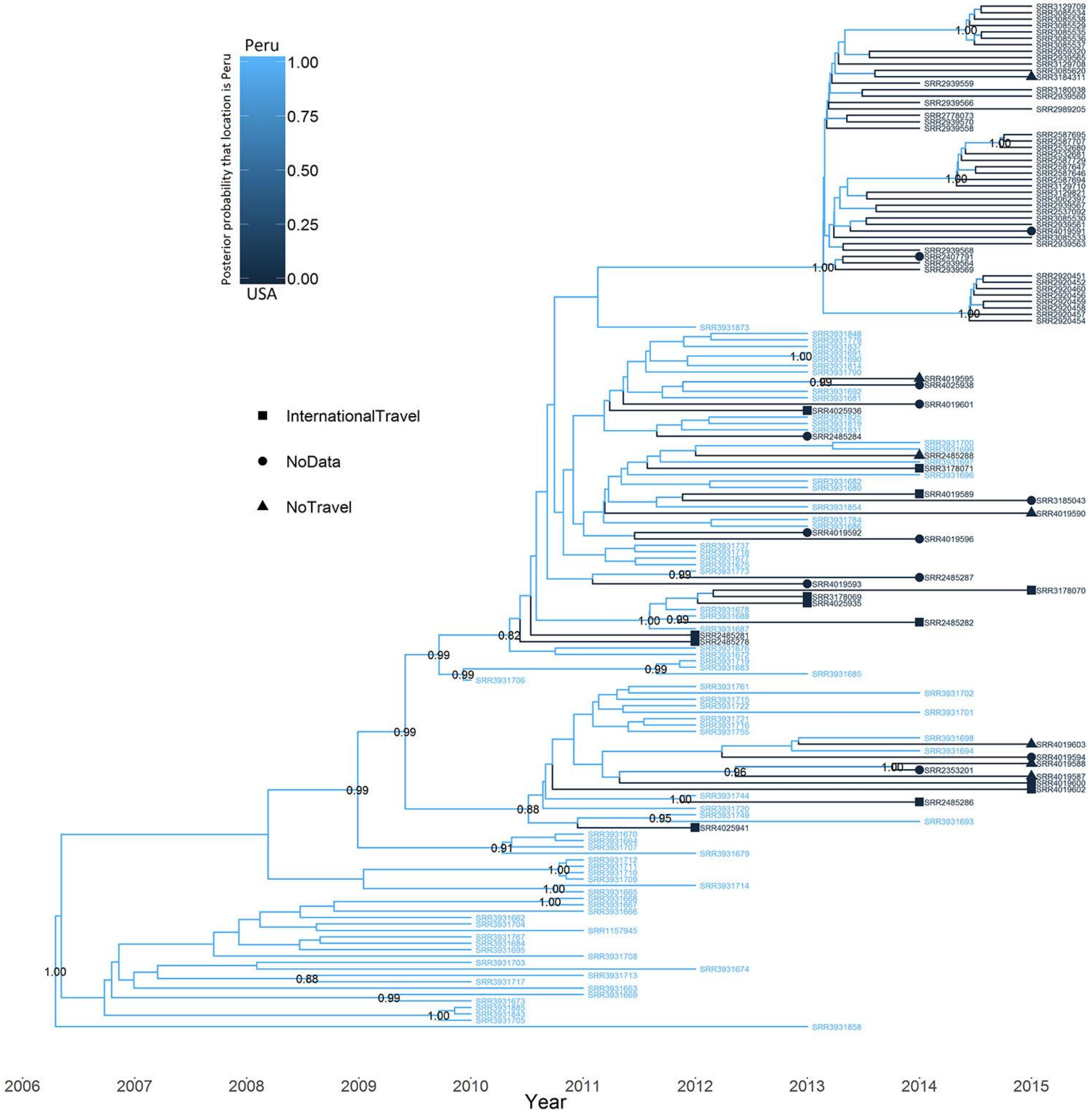

Technical Appendix Figure 1. Tip-dated maximum clade credibility tree of 160 isolates from SNP cluster PDS000003955.192, including 32 from the present study, generated using the MASCOT package in BEAST. x-axis denotes calendar year and the tree tips aligned with the year of collection for a given isolate. The tree is colored based on the posterior probability that location is Peru, with locations likely to be Peru colored light blue, and locations likely to be USA colored dark blue. Tip labels correspond with NCBI SRR ID, are colored based on the location the isolate was collected from (Peru= light blue; USA =dark blue), and symbols next to these labels indicate whether or not these isolates were from patients

with a history of international travel for isolates sequenced as part of the present study. Posterior support for internal nodes are labeled where values are >0.80.

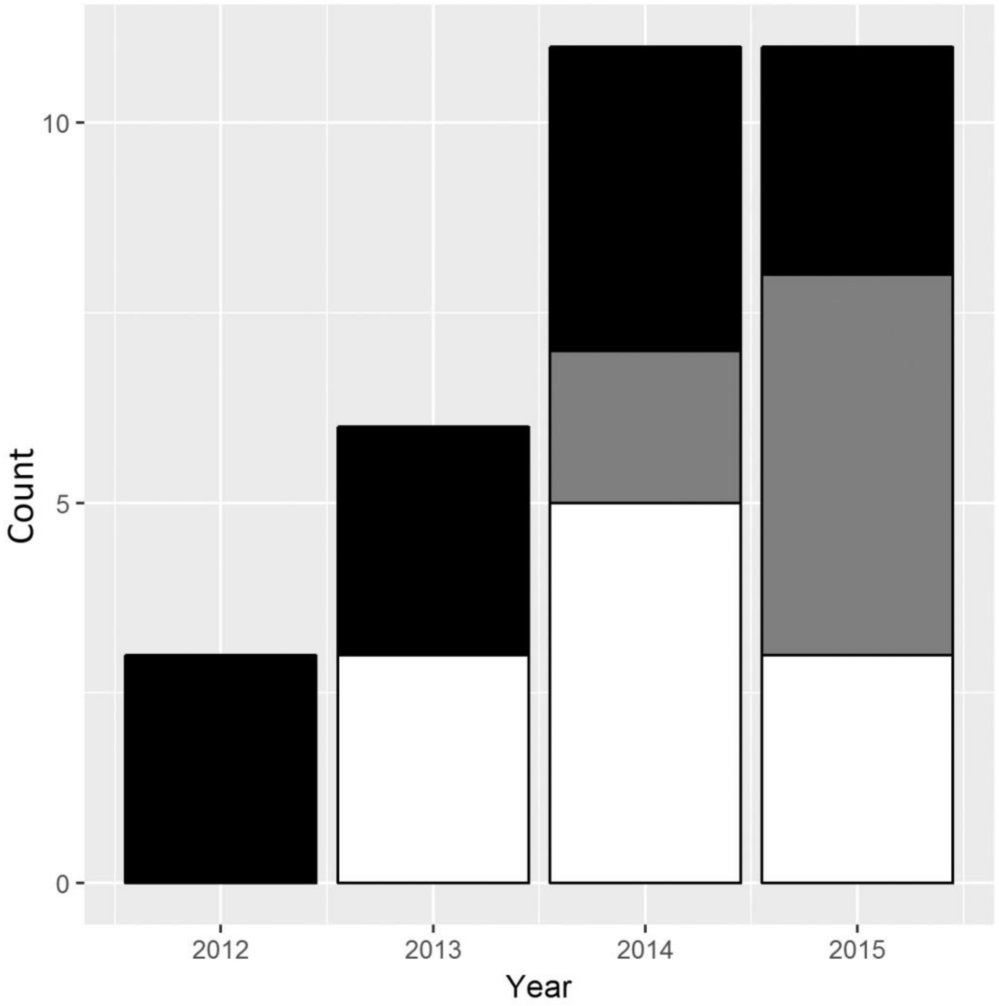

Technical Appendix Figure 2. Dates of specimen collection for isolates in Clade A, by year and history of international travel—United States, 2012–2015. International travel history denoted by shading: black indicates recent international travel; gray indicates no recent international travel; white indicates travel data are missing or not applicable.
